# Cost-effectiveness of pneumococcal vaccination strategies for the elderly in Korea

**DOI:** 10.1371/journal.pone.0177342

**Published:** 2017-05-12

**Authors:** Jung Yeon Heo, Yu Bin Seo, Won Suk Choi, Jacob Lee, Ji Yun Noh, Hye Won Jeong, Woo Joo Kim, Min Ja Kim, Hee Young Lee, Joon Young Song

**Affiliations:** 1 Division of Infectious Diseases, Department of Internal Medicine, Chungbuk National University College of Medicine, Cheongju, Republic of Korea; 2 Division of Infectious Diseases, Department of Internal Medicine, Chungbuk National University Hospital, Cheongju, Republic of Korea; 3 Division of Infectious Diseases, Department of Internal Medicine, Hallym University College of Medicine, Chuncheon, Republic of Korea; 4 Division of Infectious Diseases, Department of Internal Medicine, Korea University College of Medicine, Seoul, Republic of Korea; 5 Center for Preventive Medicine and Public health, Seoul National University Bundang Hospital, Seongnam, Republic of Korea; Azienda Ospedaliera Universitaria di Perugia, ITALY

## Abstract

**Background:**

Although the 13-valent pneumococcal conjugate vaccine (PCV13) showed good efficacy against pneumococcal disease in the the CAPiTA trial, the 23-valent pneumococcal polysaccharide vaccination (PPSV23) program has been ongoing for older adults aged ≥ 65 years in Korea since May of 2013. This study aimed to evaluate the cost-effectiveness of the current vaccination strategy (a single-dose PPSV23 vaccination) compared to a single-dose PCV13 vaccination and sequential PCV13-PPSV23 vaccinations in the elderly population aged ≥ 65 years.

**Methods:**

Using a Markov model, the incremental cost-effectiveness ratios (ICERs) of three vaccination strategies were assessed in a societal context. The transition probabilities, utility weights to estimate quality adjusted life year (QALY), and disease treatment costs were either calculated or cited from published data and the Health Insurance Review and Assessment Service. Simulations were performed in hypothetical cohorts of Korean adults aged ≥ 19 years. The vaccine effectiveness of PPSV23 was cited from a Cochrane Review report, while PCV13 effectiveness data were gathered from the CAPiTA trial.

**Results:**

Current PPSV23 vaccination strategies were cost-effective (ICER, $25,786 per QALY). However, the administration of PCV13 as a substitute for PPSV23 was shown to be more cost-effective than PPSV23 vaccination (ICER, $797 per QALY). Sequential PCV13-PPSV23 vaccination was also more cost-effective than PPSV23 for elderly people aged ≥ 65 years. In sensitivity analysis assuming significant PPSV23 effectiveness (50%) against non-bacteremic pneumococcal pneumonia, the PCV13 vaccination strategy was superior to the PPSV23 vaccination strategy in terms of cost-effectiveness.

**Conclusion:**

The results suggest that PCV13 vaccination is more cost-effective in elderly subjects aged ≥ 65 years compared to the current PPSV23 vaccination strategy. When complete data is obtained in 2018 on the maximal herd effects of childhood PCV13 immunization, the incidence of pneumococcal pneumonia and the cost-effectiveness of vaccination strategies need to be reassessed.

## Introduction

*Streptococcus pneumoniae* (Pneumococcus) is the most common bacterial pathogen in community-acquired pneumonia (CAP). It causes pneumonia as well as invasive pneumococcal disease (IPD) in adults, which is defined as the isolation of *Streptococcus pneumoniae* from a normally sterile site, resulting in high morbidity and mortality depending on age and risk group. For this reason, the 23-valent pneumococcal polysaccharide vaccine (PPSV23) has been recommended since the early 1980s for the prevention of pneumococcal disease among the elderly in many developed countries. Previous meta-analyses have shown that PPSV23 provides a considerable protective efficacy of 50–80% against IPD [1]. In previous research, however, PPSV23 did not show statistically significant protection against IPD in people aged 75 years or older or in subjects with chronic medical diseases [1]. Moreover, even though some studies have shown favorable results, PPSV23 effectiveness against non-bacteremic pneumococcal pneumonia (NPP) was not statistically significant in some existing meta-analyses [2–4].

A recent large randomized placebo-controlled trial for the 13-valent pneumococcal conjugate vaccine (PCV13), known as the CAPiTA study, demonstrates that the vaccine efficacy of PCV13 is 45.6% against vaccine-type pneumococcal CAP and 75% against vaccine-type IPD [5]. In 2014, the US Advisory Committee on Immunization Practices (ACIP) modified its guidelines to recommend sequential administration of both PCV13 and PPSV23 vaccinations for all adults aged 65 years or older on the basis of the CAPiTA study [6]. In most European countries, however, PCV13 vaccination is recommended only for high-risk immunocompromised patients due to the risk of infection due to underlying diseases [7]. This difference between the US and European countries is primarily the result of analyses on cost-effectiveness of the two types of pneumococcal vaccines, PPSV23 and PCV13 [8–11]. The differing results in cost-effectiveness studies across countries are attributable to discrepancies in the disease burden of pneumococcal pneumonia by country and to the efficacy of pneumococcal vaccines according to relevant vaccine formulation. Consequently, analysis of cost-effectiveness is required for each country to determine its national immunization policies because there are differences across countries in the incidence of pneumococcal pneumonia, hospitalization rates, and medical costs.

The National Immunization Program (NIP), which provides free PPSV23 vaccination, was implemented in May of 2013 for all people aged 65 years or older in South Korea. Together with the NIP for older adults, private vaccination with PCV10 or PCV13 has been widely implemented in children since March of 2010, reaching about 65% coverage rates (including a three-dose infant series at 68.2% coverage and a one-dose vaccination for toddlers at 62.1% coverage) [12]. By May of 2014, pediatric PCV10/PCV13 was included in the NIP, ensuring that all children are able to receive pneumococcal vaccinations. With increasing herd immunity from childhood PCV13 immunization, the incidence of IPD and pneumococcal pneumonia is also expected to gradually decrease in adults [13]. Although PCV13 has been shown to be effective against NPP based on the CAPiTA trial [5], the relative benefits from adult PCV13 vaccination may be weakened by the high level of indirect herd effects. Thus, it is necessary to review whether to maintain or to modify the vaccination policies of the NIP for older adults via analysis of cost-effectiveness of different vaccination strategies. This study aims to evaluate the cost-effectiveness of a single-dose PCV13 vaccination and sequential PCV13-PPSV23 vaccination in comparison to a single-dose PPSV23 vaccination; and of single-dose PCV13 vaccination and single-dose PPSV23 in comparison to no vaccination.

## Methods

Using a probabilistic Markov model, this study primarily intended to evaluate three pneumococcal vaccination strategies in Korean adults aged 65 years or older: (1) PPSV23 vaccination only, (2) PCV13 vaccination only, and (3) sequential PCV13-PPSV23 vaccination. In elderly subjects aged 65 years or older, targeted vaccine coverage was assumed to be either 60% or 80%, considering PPSV23 (60%) and influenza (80%) vaccine uptake rates in South Korea [14, 15]. Secondly, we estimated the cost-effectiveness of two vaccination strategies (PPSV23 versus PCV13) in adults aged 50–64 years.

We conducted a literature survey to collect input data for the analysis of cost-effectiveness. Data on local epidemiology, prognosis, and healthcare costs of pneumococcal disease in Korea were collected from the PubMed database, the KoreaMed database, annual governmental statistical reports, and the Health Insurance Review and Assessment Service (HIRA) database. Because of insufficient evidence from Korean literature, data on pneumococcal vaccine effectiveness and health state utility weights to estimate quality adjusted life years (QALYs) were obtained from the Cochrane Database of Systematic Reviews, the CAPiTA trial, and the National Health Service (NHS) Economic Evaluation Database (EED).

Using the Markov model, the incremental cost-effectiveness ratios (ICERs) of three vaccination strategies were assessed across different age and risk groups in a societal context. The ICER was calculated by dividing the net cost difference between two vaccination strategies by either the life years gained (LYG) or QALY gained by subjects. Based on the Korean gross domestic product (GDP), a gain of $25,000/QALY (< 1 × GDP per capita) was considered highly cost-effective, and a gain of $38,000/QALY (< 1.5 × GDP per capita) was accepted as moderately cost-effective[16]. Costs and benefits were discounted at a rate of 5%, converting costs to US dollars based on the value of the dollar in 2015[17].

### Markov model

**Fig 1:**
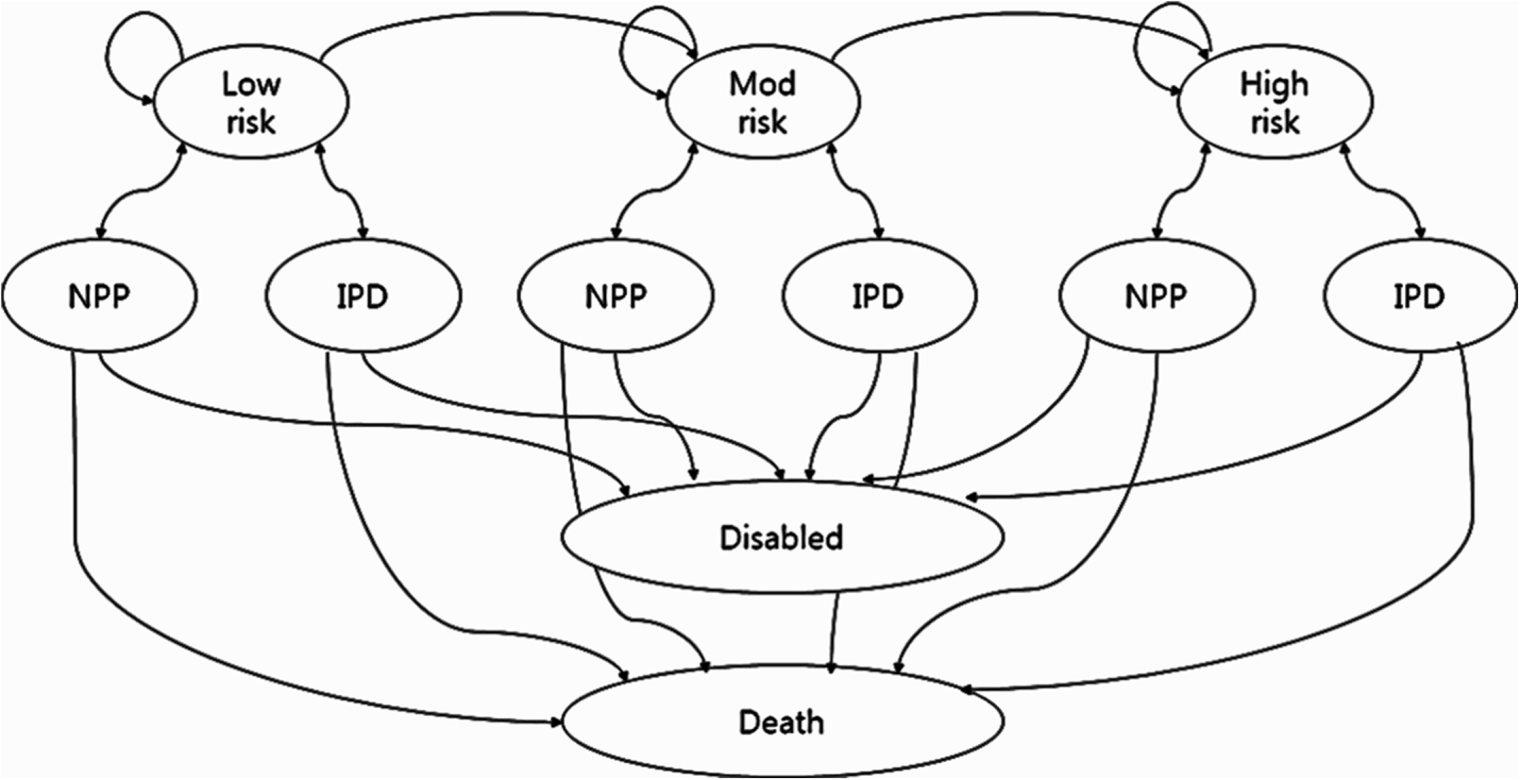
Schematic description of the Markov model (IPD, invasive pneumococcal disease; NPP, non-bacteremic pneumococcal pneumonia).

The Markov model was constructed based on epidemiological data, vaccine effectiveness, and economic parameters according to a review of previous studies (Fig 1) [8–10, 18]. Five health states were modeled, comprising health (without any pneumococcal disease), invasive pneumococcal disease, non-bacteremic pneumococcal pneumonia, neurological sequelae, and death. The probability of transition was assumed according to the age and underlying medical conditions of subjects. Vaccine adverse effects were not considered. Simulations were performed in hypothetical cohorts of Korean adults aged 19 years or older. The Markov model was set at a cycle length of one year with a 15-year time horizon following vaccination in four age cohorts (19–49, 50–64, 65–74, ≥ 75 years). The model considered persons who survived after the time horizon age with an average life expectancy based on the Korean population. Three risk groups for pneumococcal disease were defined based on underlying medical conditions as previously reported, and the subjects of each risk group had room to progress to moderate-risk or high-risk groups during follow up [19]. The proportions of the three risk groups were estimated based on the HIRA database [20].

### Definitions

Invasive pneumococcal disease (IPD) was defined as the isolation of *S*. *pneumoniae* from a normally sterile site, such as blood, cerebrospinal fluid (CSF), and pleural or ascites fluid.

As previously described, we defined NPP cases as the isolation of *S*. *pneumoniae* from lower respiratory specimens or a positive urinary antigen test for *S*. *pneumoniae* in patients with community acquired pneumonia without bacteremia [21]. As for the risk groups, high risk was defined as the presence of one or more of the following: (1) splenic dysfunction including post-splenectomy status, (2) hematologic malignancy such as multiple myeloma, leukemia, or lymphoma, (3) a condition affecting the bone marrow or lymphatic system, such as chemotherapy with alkylating drugs or antimetabolites, or radiation within the previous three months, (4) solid organ or stem cell transplantation, (5) chronic renal disease such as nephrotic syndrome or chronic renal failure, (6) HIV infection, (7) high-dose corticosteroid use (≥ 20 mg/day of prednisone or an equivalent) lasting two or more weeks, or (8) treatment with a recombinant human immunomodulator, such as rituximab, adalimumab, or infliximab. Moderate risk was defined by one or more of the following: (1) diabetes mellitus, (2) chronic liver disease, (3) chronic pulmonary disease, such as asthma or chronic obstructive lung disease, or (4) chronic cardiovascular disease, such as heart failure, cardiomyopathy, or other chronic conditions affecting cardiac function. Low risk was assigned to conditions that did not satisfy the criteria for high or moderate risk conditions.

### Clinical inputs and assumptions

Age-specific incidence rates (for IPD and NPP) were estimated using the catchment population between 2011 and 2014 (Table 1) [22]. During the period of study, annual incidence rates did not remarkably decrease. To determine risk group-specific incidence rates, we applied the ratio of each risk group versus the general population in the incidence rates of community-acquired pneumonia from the HIRA database [20]. As for case-fatality rates, age and risk group-specific IPD case-fatality rates and the proportion of patients with neurological sequelae (meningitis) were obtained from a multi-center study published by Song et al. [19]. NPP case-fatality rates were derived from a multi-center catchment population study [22]. For data with inconsistent results, we conducted a two-round modified Delphi survey with nine experts, comprising seven infectious disease specialists and two pulmonologists. The Delphi survey was conducted anonymously according to National Evidence-based Healthcare Collaborating Agency (NECA) guidelines [23]. Through the Delphi method, we assumed the case-fatality rates of NPP were 1.5-fold higher in the moderate-risk group and 2.0-fold higher in the high-risk group in comparison to the general population (i.e., the low-risk group). The average length of an IPD-related hospital stay was obtained from the multi-center study by Song et al. [19], while the average length of an NPP-related hospital stay was derived from a study by Yoo et al. with adjustment for age and risk groups [24]. In order to estimate QALY gain or loss, utility weights for health states in the general population primarily used data from the Korea National Health and Nutrition Examination Survey (KNHANES). The utility weights based on development of IPD and NPP were applied to a modified value from a study by Smith et al., reflecting the hospitalization period for IPD and NPP from a Korean study (Table 2) [8].

**Table 1:** Input data for the cost-effectiveness model of pneumococcal disease burden.

Parameters	Estimated/assumed value			
**Distribution of population age, years (%) [20]**				
19–49 (low, moderate, high risk)	86.5	10.0	3.5	
50–64 (low, moderate, high risk)	70.6	24.6	4.8	
65–74 (low, moderate, high risk)	54.6	38.2	7.2	
≥ 75 (low, moderate, high risk)	56.0	37.3	6.7	
**Incidence rate of IPD (per 100,000 persons) [22]**				
19–49 (low, moderate, high risk)	0.9	2.2	4.1	
50–64 (low, moderate, high risk)	4.3	10.9	20.5	
65–74 (low, moderate, high risk)	17.6	19.4	44.1	
≥ 75 (low, moderate, high risk)	64.3	52.8	100.9	
**Incidence rate of NPP (per 100,000 persons) [22]**				
19–49 (low, moderate, high risk)	4.9	12.3	23.3	
50–64 (low, moderate, high risk)	30.5	76.8	145.7	
65–74 (low, moderate, high risk)	213.8	237.9	539.9	
≥ 75 (low, moderate, high risk)	710.5	578.4	1112.9	
**Pneumococcal serotype coverage rate (%) [25]**				
IPD (19–49, 50–64, ≥ 65 years)				
PPSV23	59.5	59.5	65.5	
PCV13	35.1	35.1	38.1	
PPSV23 + PCV13	60.8	60.8	65.5	
NPP (19–49, 50–64, ≥ 65 years)				
PPSV23	49.8	49.8	54.9	
PCV13	35.2	35.2	38.2	
PPSV23 + PCV13	53.4	53.4	57.5	
**Sequelae, proportion (%) [19]**				
IPD (19–49, 50–64, 65–74, ≥ 75 years)	3.26	1.32	1.28	1.28
NPP (19–49, 50–64, 65–74, ≥ 75 years)	1.63	0.66	0.64	0.64
**Case-fatality rate of IPD (%) [19]**				
19–49 (low, moderate, high risk)	13.6	21.6	25.9	
50–64 (low, moderate, high risk)	22.4	25.7	30.8	
65–74 (low, moderate, high risk)	29.2	29.2	40.0	
≥ 75 (low, moderate, high risk)	37.7	37.7	72.0	
**Case-fatality rate of NPP (%) [22]**				
19–49 (low, moderate, high risk)	4.5	6.8	9.0	
50–64 (low, moderate, high risk)	7.2	10.8	14.4	
65–74 (low, moderate, high risk)	11.6	17.4	23.2	
≥ 75 (low, moderate, high risk)	14.1	21.2	28.2	
**Average length of hospital stay, IPD (days) [19]**				
19–49 (low, moderate, high risk)	19.0	15.3	22.0	
50–64 (low, moderate, high risk)	21.9	17.7	21.7	
65–74 (low, moderate, high risk)	19.9	19.0	17.3	
≥ 75 (low, moderate, high risk)	15.4	19.0	19.4	
**Average length of hospital stay, NPP (days) [24]**				
19–49 (low, moderate, high risk)	9.0	13.0	18.0	
50–64 (low, moderate, high risk)	9.0	13.0	18.0	
65–74 (low, moderate, high risk)	12.0	18.0	24.0	
≥ 75 (low, moderate, high risk)	13.0	19.0	26.0	
**Direct medical costs per case, IPD (US$) [19]**				
19–49 (low, moderate, high risk)	6,849	5,404	6,990	
50–64 (low, moderate, high risk)	8,528	8,756	7,135	
65–74 (low, moderate, high risk)	8,557	6,964	8,609	
≥ 75 (low, moderate, high risk)	8,090	5,730	7,880	
**Direct medical costs per case, NPP (US$) [20]**				
19–49 (low, moderate, high risk)	1,055	2,234	2,986	
50–64 (low, moderate, high risk)	1,406	2,624	2,871	
65–74 (low, moderate, high risk)	1,701	2,651	2,800	
≥ 75 (low, moderate, high risk)	1,812	2,390	2,538	

IPD, invasive pneumococcal disease; NPP, non-bacteremic pneumococcal pneumonia.

**Table 2:** The utility weights by age and risk groups and pneumococcal disease.

Age group	Risk	Health-state utility	Utility by acute event	
		General population	IPD	NPP
18–49 years	Low	0.972	0.0416	0.0197
	Moderate	0.972	0.0335	0.0285
	High	0.843	0.0482	0.0395
50–64 years	Low	0.948	0.048	0.0197
	Moderate	0.948	0.0388	0.0285
	High	0.792	0.0476	0.0395
65–74 years	Low	0.913	0.0436	0.0263
	Moderate	0.913	0.0416	0.0395
	High	0.682	0.0379	0.0526
≥75 years	Low	0.85	0.0338	0.0285
	Moderate	0.85	0.0416	0.0416
	High	0.68	0.0425	0.057

Serotype distribution of *S*. *pneumoniae* was estimated during early periods (2013–2015 years) after introduction of PPSV23 NIP for the elderly [25]. In old adults aged ≥65 years, the vaccine serotypes for IPD were 65.5% for PPSV23 and 38.1% for PCV13 (Table 1). For NPP, those values were 54.9% and 38.2%, respectively. As shown in Table 3, the vaccine effectiveness of PPSV23 was cited from a Cochrane Review report, while PCV13 effectiveness data was obtained from the CAPiTA trial [4, 5]. Meta-analysis showed that the effectiveness of PPSV23 for serotype-specific IPD was estimated to be about 82% (69%-90%) in adults, while its effectiveness in reducing non-bacteremic pneumococcal pneumonia (NPP) was inconsistent [3, 4, 26]. Accordingly, based on the literature review, we conducted the Delphi survey and assumed the base effectiveness of PPSV23 in preventing NPP to be 0%. The effectiveness of PPSV23 against IPD was age-adjusted in proportion to those in the study by Smith et al. [8]. The effectiveness of PPSV23 against IPD was assumed to be 20% lower among patients with chronic medical conditions (the moderate-risk group) and was considered to be even less effective (base 20%) in immunocompromised patients (the high-risk group) [1, 27, 28]. The effectiveness of PCV13 in reducing vaccine-serotype IPD and NPP in low-risk patients was established as 75.0% and 45.0%, respectively, according to findings from the CAPiTA trial [5]. Based on a previous report, the relative effectiveness of PCV13 against IPD was estimated to be 15% and 22% lower in moderate-risk and high-risk groups in comparison to the low-risk group, respectively [29]. Similarly, based on the literature review and Delphi survey, PCV13 effectiveness against NPP was estimated to be 20% and 35% lower in moderate-risk and high-risk groups in comparison to the low-risk group, respectively [29]. The effectiveness of both PPSV23 and PCV13 was assumed to decrease with age at vaccination and by the number of years that had elapsed since vaccination. This decrease in effectiveness was established in proportion to the extent of detriment demonstrated in the study by Smith et al. (Table 3) [8]. Serotype distribution of *S*. *pneumoniae* was estimated during early periods (2013–2015 years) after introduction of PPSV23 NIP for the elderly [25]. In old adults aged ≥65 years, the vaccine serotypes for IPD were 65.5% for PPSV23 and 38.1% for PCV13 (Table 1). Those values for NPP were 54.9% and 38.2%, respectively.

**Table 3:** Estimates of PPSV23 and PCV13 vaccine effectiveness (VE) against invasive pneumococcal disease and non-bacteremic pneumococcal pneumonia.

VE against invasive pneumococcal disease (%) [4, 5, 8]						
	PPSV23			PCV13		
Years post-vaccination	< 65 years	65–74 years	≥ 75 years	< 65 years	65–74 years	≥ 75 years
	Base (low-high)	Base (low-high)	Base (low-high)	Base (low-high)	Base (low-high)	Base (low-high)
1	95.3 (92.0–95.0)	82.0 (69.0–90.0)	68.7 (23.0–85.0)	79.4 (61.7–88.2)	75.0 (52.9–83.8)	62.8 (17.6–19.2)
3	91.2 (85.1–94.5)	74.8 (57.5–83.0)	54.3 (0–83.5)	70.6 (48.5–83.8)	70.6 (39.7–79.4)	51.2 (13.2–75.0)
5	87.2 (76.0–90.0)	59.5 (35.1–80.0)	32.8 (0–75.0)	61.8 (44.2–79.4)	61.8 (26.5–76.8)	34.1 (8.8–72.5)
7	61.5 (46.2–75.0)	33.8 (15.0–48.0)	10.3 (0–30.0)	52.9 (33.2–70.8)	52.9 (19.9–68.4)	16.0 (6.6–64.6)
10	20.5 (0–30.0)	0 (0–10.0)	0 (0–10.0)	44.1 (35.2–70.6)	44.1 (13.2–60.0)	0 (0–56.7)
15	0 (0–20.0)	0 (0–10.0)	0 (0–10.0)	39.7 (0–52.9)	29.1 (0–52.9)	0 (0–50.0)
VE against non-bacteremic pneumococcal pneumonia (%) [3–5, 8]						
	PPSV23			PCV13		
Years post-vaccination	< 65 years	65–74 years	≥ 75 years	< 65 years	65–74 years	≥ 75 years
	Base (low-high)	Base (low-high)	Base (low-high)	Base (low-high)	Base (low-high)	Base (low-high)
1	-	-	-	52.0 (37.1–52.6)	45.0 (31.8–50.3)	37.7 (26.6–42.1)
3	-	-	-	46.6 (29.1–50.5)	42.4 (23.8–47.6)	30.7 (17.3–34.6)
5	-	-	-	39.9 (26.5–47.9)	37.1 (15.9–46.1)	20.4 (8.8–25.4)
7	-	-	-	34.2 (19.8–42.6)	31.8 (11.9–41.0)	9.6 (3.6–12.4)
10	-	-	-	28.6 (21.1–42.5)	26.5 (7.9–36.0)	0 (0–4.1)
15	-	-	-	25.9 (0–31.8)	17.5 (0–31.8)	0 (0–4.1)

PPSV23, 23-valent pneumococcal polysaccharide vaccine; PCV13, 13-valent pneumococcal conjugate vaccine

Indirect effects from childhood PCV13 immunization were extrapolated from the effects in subjects after PCV7 vaccination [30, 31]. As previously reported, an uptake rate of 65–75% of childhood vaccination is required to induce herd protection [31, 32]. In the United States, documented indirect effects of PCV13 are presented within two years of use with increasing vaccine uptake rates [33]. Similarly, in Korea, the PCV13 uptake rate in children aged three years or younger was estimated to be around 65% in 2012, since the introduction of the vaccine in 2010 [12]. Therefore, from Moore el al., we assumed that the indirect effects of IPD following PCV13 vaccination have gradually increased over the years since 2012, with the maximal steady level to be reached within seven years in 2018 (Table 4) [33]. Based on our findings from the Delphi survey, we presumed the potential indirect PCV13 effects of NPP at half the level of the indirect PCV13 effects of IPD.

**Table 4:** Assumption of indirect effects against invasive pneumococcal disease from childhood PCV13 immunization.

Assumed indirect effects against invasive pneumococcal disease from childhood immunization (%) [30, 31, 33]			
Year	19–49 years	50–64 years	≥ 65 years
2014	32.0	18.0	12.0
2015	39.7	22.3	14.9
2016	46.9	26.4	17.6
2017	50.8	28.5	19.0
2018	55.2	31.0	20.7

PCV13, 13-valent pneumococcal conjugate vaccine

### Costs

Direct healthcare costs for IPD and NPP were derived from the work of Song et al. and from an unpublished study using the HIRA database [19, 20]. Non-healthcare costs included the costs of caregiver time ($66.7 per day) and transportation expenses ($32), which were calculated based on the KNHANES report [34]. For patients younger than 65 years, indirect non-healthcare costs (productivity loss) were calculated by multiplying the patient’s average daily wages and work loss days (length of hospital stay). Average daily wages were derived from statistics of employment and labor and were set at $59.2 for adults aged 19–49 years and $64.0 for adults aged 50–64 years.

For vaccine costs, we applied governmental contract prices for both PPSV23 ($14.13) and PCV13 ($50.31) as of 2015. In addition, a vaccine administration fee of $15 was considered. All costs are expressed in US dollars (1 US$ = 1,200 KRW) [35]. Data are shown in Table 1.

### Sensitivity analysis

**Fig 2:**
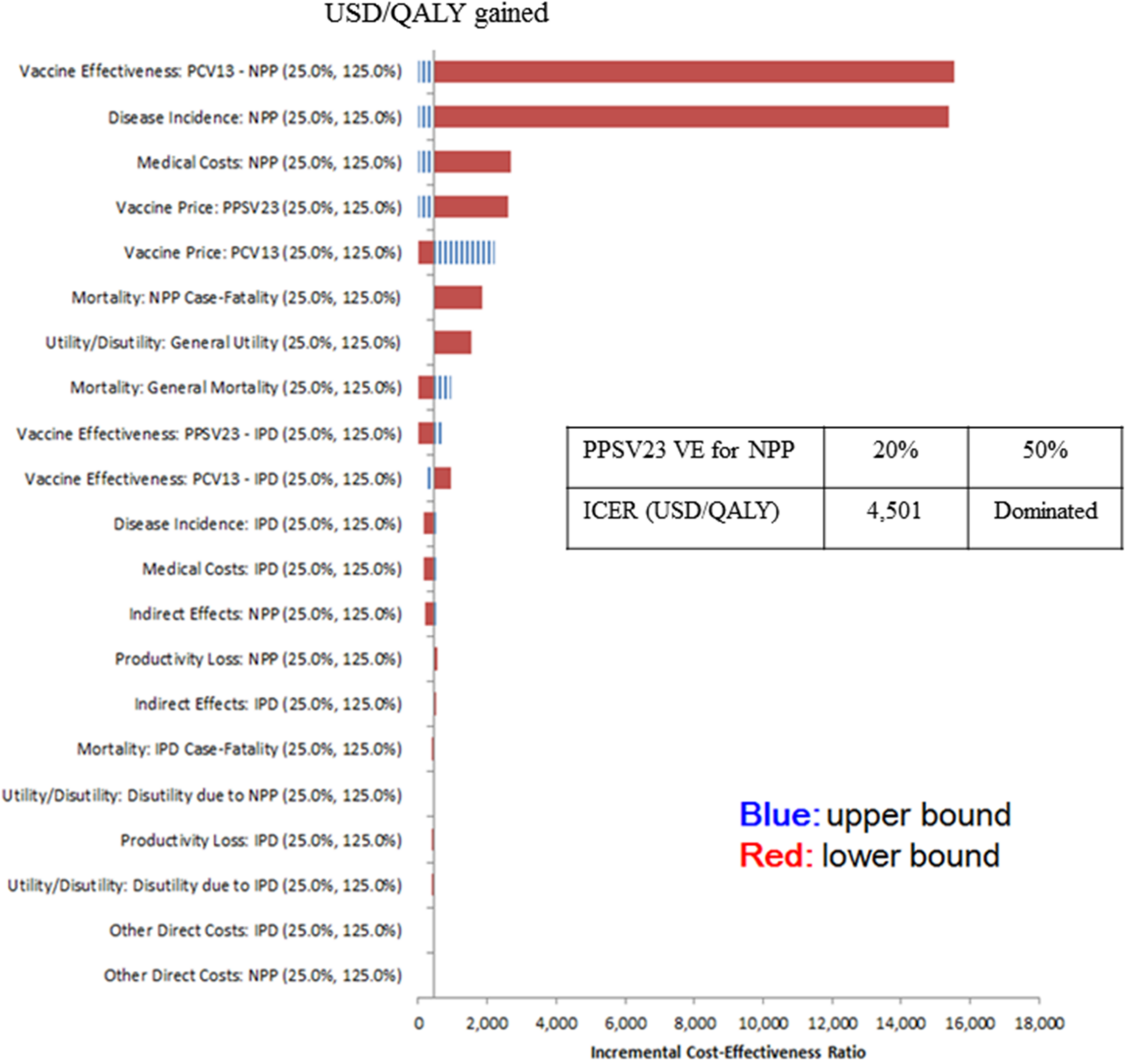
Results of one-way sensitivity analysis: PCV13 versus PPSV23. **The base value of PPSV23 effectiveness against NPP was assumed as 0%, and sensitivity analysis was conducted by varying the value from 20% to 50%, as presented in the 2x2 table.** (PPSV23, 23-valent pneumococcal polysaccharide vaccine; PCV13, 13-valent pneumococcal conjugate vaccine; IPD, invasive pneumococcal disease; NPP, non-bacteremic pneumococcal pneumonia).

We performed one-way and probabilistic sensitivity analyses comparing the PCV13 strategy with current PPSV23 strategies in elderly subjects aged 65 years or older. One-way sensitivity analysis was conducted by separately increasing/decreasing each input parameter by 25% while keeping other parameters constant. For PPSV23 effectiveness against NPP, we assumed the base value as 0% and varied it from 20% to 50% in sensitivity analyses (Fig 2). The parameters and percentage of variation were determined by expert opinion based on literature review. Probabilistic analysis was performed using a Monte Carlo simulation with 1,000 interactions, with all of the input parameter values selected from a probability distribution. A triangular distribution was used because of the limited distribution information.

## Results

### Comparison of cost-effectiveness among three pneumococcal vaccination strategies in elderly subjects aged 65 years or older

In the findings of this study, both PPSV23 and PCV13 vaccination strategies in comparison with no vaccination were cost-effective with ICERs less than $38,000/QALY (Table 5). Compared to no vaccination, the estimated ICERs of current PPSV23 vaccination strategy were $25,786 per QALY with a targeted vaccine uptake rate of 60% and $17,354 per QALY with a targeted vaccine uptake rate of 80%. Compared to no vaccination, the ICERs for PCV13 vaccination were $4,529 per QALY and $5,045 per QALY with 60% and 80% targeted vaccine uptake rates, respectively. However, the administration of PCV13 as a substitute for the current PPSV23 strategy was more cost-effective than PPSV23 vaccination, with results showing that ICERs were $797 per QALY and $701 per QALY with 60% and 80% targeted vaccine uptake rates, respectively. The strategy of PCV13 vaccination was cost-effective compared to that of PPSV23 vaccination alone for elderly subjects aged 65 years or older, regardless of the age and risk groups (Table 6). Sequential PCV13-PPSV23 vaccination was also cost-effective in comparison to vaccination with only PPSV23 for elderly subjects aged 65 years or older, regardless of vaccine uptake rate (ICER, $1,228 per QALY at a 60% vaccination rate; ICER, $10,645 per QALY at an 80% vaccination rate).

**Table 5:** Analyses of cost-effectiveness according to vaccination strategy.

Strategies	Cost per patient	Incremental cost	Effectiveness per patient	Incremental effectiveness per patient	ICER*
	$	$	QALY	QALY	$/QALY
No vaccination	53.71	-	12.2000	-	-
PPSV23 for elderly aged ≥65 y (vaccine uptake rate of 60%)	56.53	2.83	12.2001	0.00011	25,786
PCV13 for elderly aged ≥65 y (vaccine uptake rate of 60%)	58.19	4.48	12.2010	0.00099	4,529
PPSV23 for elderly aged ≥65 y (vaccine uptake rate of 80%)	56.82	3.12	12.2002	0.00018	17,354
PCV13 for elderly aged ≥65 y (vaccine uptake rate of 80%)	59.27	5.55	12.2010	0.0011	5,045
Current strategy†	56.53	-	12.2000	-	-
PCV13 for elderly aged ≥65 y (vaccine uptake rate of 60%)	57.10	0.56	12.2008	0.0007	797
PCV13 for elderly aged ≥65 y (vaccine uptake rate of 80%)	57.17	0.63	12.2010	0.0009	701
Sequential PCV13-PPSV23 for elderly aged ≥65 y (vaccine uptake rate of 60%)	57.14	0.61	12.2005	0.0005	1,228
Sequential PCV13-PPSV23 for elderly aged ≥65 y (vaccine uptake rate of 80%)	59.73	3.19	12.2003	0.0003	10,645

* ICER: incremental cost-effectiveness ratio ($/QALY gained)

†Current strategy: PPSV23 vaccination for elderly aged ≥65 years with a targeted uptake rate of 60%

**Table 6:** Analyses of incremental cost-effectiveness ratios (ICERs) based on age and risk groups.

Strategies	Age group (years)	Low-risk group	Moderate-risk group	High-risk group
		ICER ($/QALY)		
PCV13 versus no vaccination	50–64	Dominated	16,782	7,188
	65–74	4,219	5,512	2,360
	75–84	2,632	4,269	3,243
	85–99	6,425	9,917	8,916
PCV13 versus PPSV23	50–64	Dominated	10,545	3,761
	65–74	44	8	Dominant
	75–84	Dominant	395.86	Dominant
	85–99	1,971	3,828	2,168

PPSV23, 23-valent pneumococcal polysaccharide vaccine; PCV13, 13-valent pneumococcal conjugate vaccine

### Cost-effectiveness of PCV13 in adults aged 50–64 years

Compared to no vaccination, the PCV13 vaccination strategy with a 60% targeted vaccination rate was cost-effective in the moderate-risk (ICER, $16,782 per QALY) and high-risk (ICER, $7,188 per QALY) groups, but not in the low-risk group (which dominated) (Table 6). When PCV13 versus PPSV23 strategies were compared with a targeted vaccination rate of 60%, the PCV13 vaccination strategy was also cost-effective in moderate-risk (ICER, $10,545 per QALY) and high-risk (ICER, $3,761 per QALY) groups, but not in the low-risk group (which dominated) (Table 6).

### Sensitivity analysis

**Fig 3:**
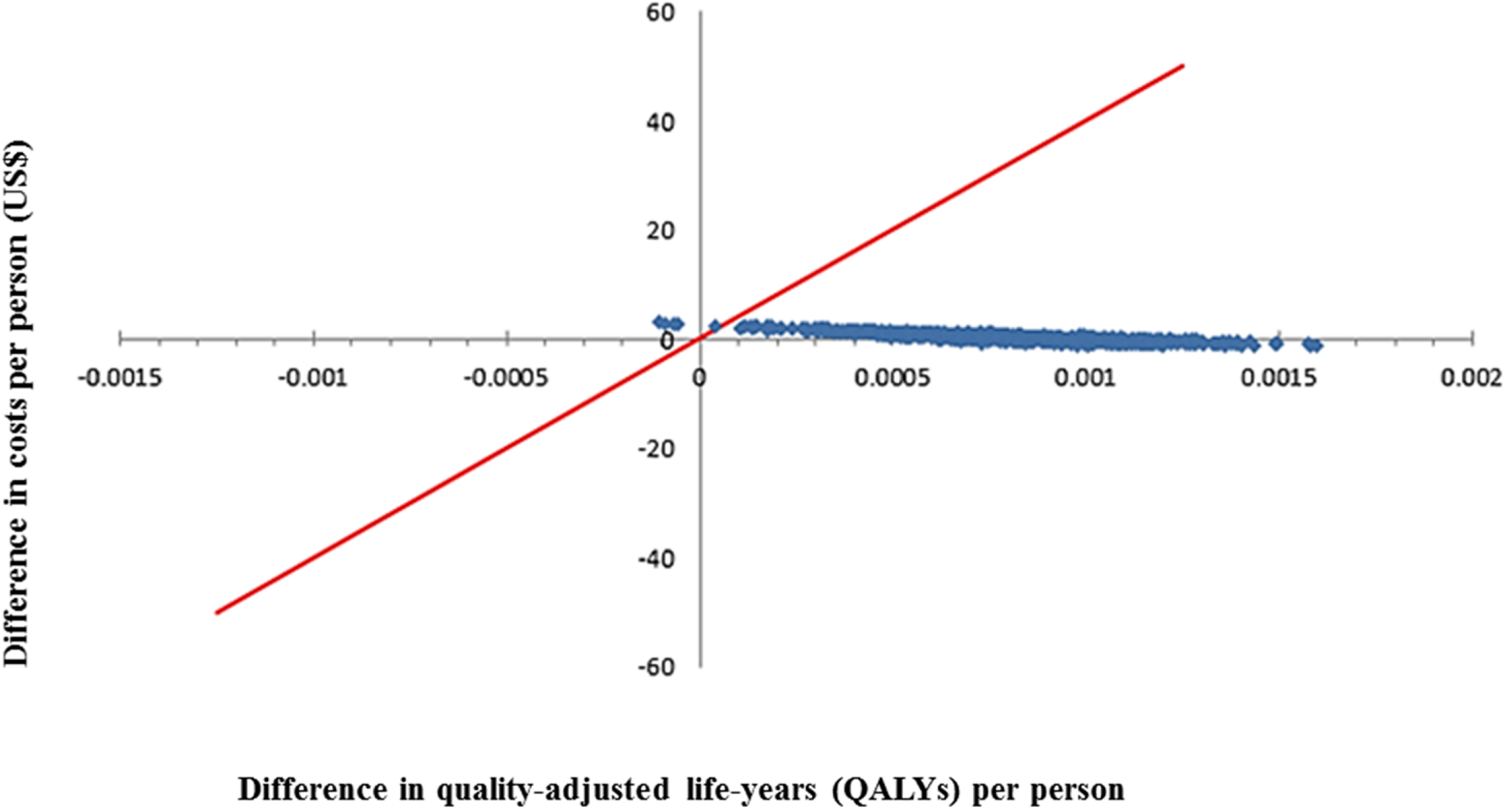
Results of probabilistic sensitivity analysis: PCV13 versus PPSV23 (PPSV23, 23-valent pneumococcal polysaccharide vaccine; PCV13, 13-valent pneumococcal conjugate vaccine).

The ICER was most sensitive to the incidence of NPP and vaccine effectiveness against NPP (Fig 2). In 25% increasing/decreasing one-way sensitivity analysis, all ICERs were within the limits of cost-effectiveness. When we assumed low-level PPSV23 effectiveness (20%) against NPP, the PCV13 vaccination strategy was still cost-effective in comparison to PPSV23 (ICER, $4,501 per QALY). However, with the assumption of significant PPSV23 effectiveness (50%) against NPP, the PCV13 vaccination strategy was shown to be inferior to the PPSV23 vaccination strategy in the elderly. Fig 3 shows the results of probabilistic analysis. Of 1,000 random simulations, 99.6% were cost-effective.

## Discussion

This study analyzes the cost-effectiveness of an alternative single-dose of PCV13 vaccination and sequential PPSV23-PCV13 vaccinations in comparison to the current single-dose strategy of PPSV23 vaccination for elderly people aged 65 years or older. In addition, the study investigates the cost-effectiveness of pneumococcal vaccination strategies for adults aged 50–64 years with respect to underlying medical conditions. This analysis shows that both PCV13 and PPSV23 vaccination strategies are more favorable than no vaccination for elderly subjects aged 65 years or older. Replacement of the current single-dose PPSV23 vaccination with a single-dose PCV13 or with sequential PPSV23-PCV13 vaccinations is also found to be cost-effective. Moreover, PCV13 vaccination is shown to be cost-effective in people aged 50–64 years with underlying diseases who are categorized as moderate- or high-risk patients. All of these results, however, are sensitive to assumptions about the pneumococcal vaccine effectiveness against NPP and the incidence of NPP. If PPSV23 is assumed to have a significant effect of 50% on NPP, then the current PPSV23 vaccination strategy is possibly favorable in comparison to PCV13 vaccination. On the other hand, the degree of PPSV23/PCV13 effectiveness against NPP and the reduction in NPP incidence are clearly correlated with increasing ICER values. These results highlight the key points that should be considered by policy makers in determining pneumococcal vaccination strategies.

Much research has reported on the cost-effectiveness of pneumococcal vaccines in the US and Europe [8–11, 36–40]. The majority of US studies report that PCV13 vaccination is cost-effective in people aged 50 years or older or 65 years or older, while European-based studies do not show consistent results [8–11, 36–40]. In each European study, the differing levels of protection against NPP according to vaccine formulation considerably impact cost-effectiveness analysis. Cost-effectiveness analyses in Germany and in the United Kingdom have shown that PPSV23 is more cost-effective than PCV13 [11, 40]. Both of these studies assume that PPSV23 could provide 39% protective effectiveness against NPP based on the results of a prospective cohort study (EVAN-65 study) in Spain, and that both PPSV23 and PCV13 have potentially comparable effectiveness in preventing IPD and NPP [11, 40, 41]. On the other hand, PCV13 vaccination is thought to be more cost-effective than PPSV23 in adults aged 50 or 65 years or more, conditional to the hypothesis that PCV13 vaccination in adults has similar preventive effectiveness against pneumonia to PCV7 vaccination in children, but PPSV23 is ineffective [8]. In a later US study by Stoecker et al., which applies the results of the CAPiTA trial, both PCV13 addition and replacement strategies are shown to be cost-effective in comparison to previous PPSV23 vaccination strategies in older adults aged 65 years or older [9]. More specifically, the ICER of a replacement strategy is supposed to increase more significantly in comparison to the ICER of PCV13 addition strategies. A recent study in Japan also evaluates the cost-effectiveness of vaccines [18]. In Japan, a PPSV23 immunization program for subjects aged 65 years or older, similar to the Korean NIP, was introduced in October of 2014. However, the eligible recipients for subsidized PPSV23 vaccination were limited to persons aged 65, 70, 75, 80, 85, 90, 95, and ≥100. According to that research, selective vaccination with either PPSV23 or PCV13 for elderly aged 65 years or older, is more cost-effective than the original Japanese strategy. This new strategy is shown to remain cost-effective when the proportion of PCV13 vaccination changes from 10% to 90% in sensitivity analysis. In England, PCV13 vaccination for children and PPSV23 vaccination programs for elderly people aged 65 years or older have been implemented. Although this situation is similar to the situation in South Korea, PCV13 vaccination for subjects aged 65 years or older is not considered cost-effective in England [10]. Due to a high degree of indirect effects from childhood PCV13 immunization, the incidence of pneumococcal disease in older adults is assumed to decrease by 40–60% from 2015–2016 to 2018–2019 [10]. The projected indirect effects in England are higher than those in the present study [10]. In addition to the reduction in incidence of pneumococcal disease, serotype replacement would progress over time, thereby impacting the cost-effectiveness of vaccination strategies. The work of Stoecker et al. projects the proportion of PCV13 serotypes at less than 4% of IPD cases in a recent report sponsored by the US Centers for Disease Control and Prevention (CDC) [9]. Thus, PPSV23 vaccination may become more cost-effective than PCV13 vaccination with the progression of serotype replacement and limited serotype coverage [42]. Several reports showed that a high level of PCV vaccination resulted in serotype replacement to a non-vaccine serotype [30, 43, 44]. Nevertheless, the degree of serotype change could be variable in different age groups and populations, in spite of serotype replacement.

There are some limitations to this study. First, there is insufficient data on the incidence of pneumococcal disease in Korean adults. Although age-specific incidence of IPD and NPP was derived from a multi-center catchment population-based study, a decreasing trend in pneumococcal disease due to herd immunity has not been observed over the period of study (2011–2014) [22]. Considering the reports from Western countries, the incidence of adult pneumococcal disease may decrease substantially in the upcoming one to three years [30, 33, 45]. These results may have a significant effect on ICER values. However, it is difficult to predict real changes in pneumococcal disease incidence. Thus, it is essential to establish a surveillance system to estimate the incidence and serotype distribution of pneumococcal disease on an ongoing basis. Second, we could not sufficiently reflect the serotype replacement in this study. In South Korea, serotype replacement is ongoing, and the level of the serotype replacement varied among age groups. As presented in Table 4, indirect effects from childhood immunization were estimated to be lower in the elderly aged ≥65 years compared to those in young adults. Eventually, the indirect effects would be more remarkable across the different age groups. Third, influenza is a leading cause of pneumococcal disease [46, 47]. Both influenza and pneumococcal vaccines show synergistic effects in preventing influenza/pneumonia-related hospitalization and death among many sample populations [48, 49]. Despite high annual influenza vaccine uptake rates (about 80% in elderly people aged 65 years or older) in South Korea, influenza vaccination is not considered in existing cost-effectiveness analysis [14]. Forth, the change of QALY and costs caused by PCV13 or PPSV23-related adverse events were not considered in our model. The pneumococcal vaccine type-specific incidence of adverse events and related costs were not available in South Korea. Previous clinical trials showed that overall systemic adverse events were comparable between both type of vaccine recipients, and serious adverse events were rare for both pneumococcal vaccine types [50, 51].

In conclusion, the current PPSV23 NIP for the elderly population in South Korea is cost-effective. However, in comparison to current PPSV23 vaccination strategies, both PCV13-addition (i.e., sequential PCV13-PPSV23 vaccination) and PCV13-replacement strategies would be more cost-effective for elderly people aged 65 years or older, as well as for chronically ill patients aged 50–64 years. In addition to cost-effectiveness, policy makers should consider a diverse set of uncertain factors, including serotype replacement, incremental indirect effects, and projected disease incidence. Based on reasonable assumptions, both age-based and risk-based approaches need to be compared with respect to the budget impact on pneumococcal disease burden. When the indirect effects from childhood PCV13 immunization peak in 2018, it will be necessary to re-evaluate the incidence of pneumococcal pneumonia and the cost-effectiveness of existing vaccination strategies.
